# Protocolo para la Telerrehabilitación del paciente amputado de miembro inferior utilizando tecnologías móviles

**DOI:** 10.15446/rsap.V25n4.105365

**Published:** 2023-07-01

**Authors:** Héctor D. Vanegas-Sáenz, Nohora E. Alvarez-Rey, Lesley F. Bohórquez-Chacón, Enith J. Pacheco-Casadiegos, Jhon E. Lizarazo-Parada

**Affiliations:** 1 HV: FTO. Esp. Fisioterapia Neurorehabilitación. Facultad de Ciencias Médicas y de la Salud, Universidad de Santander. Cúcuta, Colombia. da.vanegas@mail.udes.edu.co Universidad de Santander Fisioterapia Neurorehabilitación Facultad de Ciencias Médicas y de la Salud Universidad de Santander Cúcuta Colombia da.vanegas@mail.udes.edu.co; 2 NA: FTO. Esp. Gerencia y Auditoria de la Atención en Salud. Facultad de Ciencias Médicas y de la Salud, Universidad de Santander. Cúcuta, Colombia. no.alvarez@mail.udes.edu.co Universidad de Santander Gerencia y Auditoria de la Atención en Salud Facultad de Ciencias Médicas y de la Salud Universidad de Santander Cúcuta Colombia no.alvarez@mail.udes.edu.co; 3 LB: Ing. Sistemas. M. Sc. Dirección Estratégica. Especialidad Gerencia: Recursos Humanos y Gestión de Conocimiento. Facultad de Ingenierías, Universidad de Santander. Cúcuta, Colombia. le.bohorquez@mail.udes.edu.co Universidad de Santander Especialidad Gerencia: Recursos Humanos y Gestión de Conocimiento Facultad de Ingenierías Universidad de Santander Cúcuta Colombia le.bohorquez@mail.udes.edu.co; 4 EP: Ing. Sistemas. M. Sc. Comunicación Digital. Facultad de Ciencias Económicas, Administrativas y Contables. Universidad de Santander, Cúcuta, Colombia. en.pacheco@mail.udes.edu.co Universidad de Santander Facultad de Ciencias Económicas Administrativas y Contables Universidad de Santander Cúcuta Colombia en.pacheco@mail.udes.edu.co; 5 JL: Ing. Mecatrónico. M. Sc. Ingeniería de Mantenimiento Industrial. Servicio Nacional de Aprendizaje (SENA). Cúcuta, Colombia. jlizarazop@sena.edu.co Ingeniería de Mantenimiento Industrial Servicio Nacional de Aprendizaje Cúcuta Colombia jlizarazop@sena.edu.co

**Keywords:** Amputación, fisioterapia, telerrehabilitación, aplicaciones móviles, protocolos clínicos, tecnología, animación *(fuente: DeCS, BIREME)*, Amputation, physiotherapy, telerehabilitation, mobile applications, clinical protocols, technology, animation *(source: MeSH, NLM)*

## Abstract

**Objetivo:**

Diseñar un protocolo para la telerrehabilitación del paciente amputado de miembro inferior utilizando tecnologías móviles.

**Métodos:**

Estudio cuantitativo descriptivo. Para el diseño del protocolo se realizó la búsqueda de la literatura en bases de datos indexadas y la validación del contenido por expertos en el área. Se convocó a trece expertos en el área de la rehabilitación que evaluaron el contenido de los protocolos en términos de adecuación, pertinencia y relevancia. Posteriormente, se realizó un consenso para definir los protocolos que se usaron en el software. Se utilizó una escala tipo Likert con puntuación de 1 a 5, en la cual 1 se refiere a la más baja calificación y 5 corresponde a la más alta, puntajes que luego fueron traducidos a tres categorías donde 1 y 2 se convirtieron en "No importante", 3 y 4 se clasificaron en "Útil, no esencial", y 5 se clasificó como "Esencial", para el cálculo del índice de validez de contenido. Se estimó el coeficiente Kappa de Fleiss para cada criterio, donde para la interpretación se tuvo en cuenta la escala establecida por Landis y Koch, que expresa cualitativamente la fuerza de concordancia entre los evaluadores.

**Resultados:**

El protocolo tiene propiedades aceptables para ser utilizado como herramienta de intervención del paciente amputado de miembro inferior. Se dispuso en un software para teléfonos móviles denominado Apptivate, el cual fue diseñado mediante un trabajo interprofesional y contiene el número de repeticiones y de series que se debe realizar de cada ejercicio, con animaciones, texto y audio descriptivo.

## Abstract (en)

**Objective:**

To design a protocol for telerehabilitation of the lower limb amputee patient using mobile technologies.

**Methods:**

Quantitative descriptive study. For the design of the protocol, a literature search in indexed databases and validation of the content by experts in the field were carried out. Thirteen experts in the field of rehabilitation were convened to evaluate the content of the protocols in terms of adequacy, pertinence and relevance. Subsequently, a consensus was reached to define the protocols used in the software. A Likert-type scale with a score from 1 to 5 was used, where 1 refers to the lowest rating and 5 corresponds to the highest, scores that were then translated into three categories where 1 and 2 became "Not important", 3 and 4 were classified as "Useful, not essential", and 5 was classified as "Essential", for the calculation of the content validity index. The Fleiss Kappa coefficient was estimated for each criterion, where the scale established by Landis and Koch, which qualitatively expresses the strength of agreement between the evaluators, was taken into account for the interpretation.

**Results:**

The protocol has acceptable properties to be used as an intervention tool for lower limb amputee patients, it was provided in a software for cell phones called Apptivate, which was designed through an interprofessional work and contains the number of repetitions and series to be performed for each exercise, with animations, text and descriptive audio.

La rehabilitación del paciente amputado de miembro inferior incluye la restitución del miembro amputado y el manejo fisioterapéutico, con el propósito de que el paciente logre la máxima funcionalidad que le permita reincorporarse en los roles familiar, social y laboral 1.

Para lograr la independencia funcional de los pacientes amputados, los fisioterapeutas implementan ejercicios enfocados en potenciar las cualidades físicas del movimiento 2,3. En los países de altos ingresos, más del 60% de las personas con algún tipo de amputación han logrado con éxito una rehabilitación integral 4.

La Organización Mundial de la Salud (OMS) estimó que 650 millones de personas en todo el mundo presentan discapacidad 5, la mayoría vive en países de bajos ingresos y corresponde al 84% de la población mundial. Asimismo, el 3% de la población con discapacidad tiene acceso a los servicios de rehabilitación, aproximadamente 30 millones de personas con necesidades protésicas viven en países de bajos ingresos, tienen dificultades para acceder a los servicios de salud y por esta razón no reciben una atención temprana 6. La discapacidad varía de acuerdo con una compleja combinación de factores, entre ellos, el sexo, el ciclo de vida, la exposición a riesgos ambientales, la situación socioeconómica, la cultura y la disponibilidad de recursos 7.

En Colombia, según la Ley 1419 del 2010 8, existen lineamientos para el desarrollo de la telesalud de acuerdo con los principios de eficiencia, universalidad, solidaridad, integralidad y calidad. La telesalud hace referencia a las actividades relacionadas con la salud, los servicios y los métodos, que se llevan a cabo a distancia y con la ayuda de las tecnologías de información y comunicaciones (TIC) 9. La telerrehabilitación presta servicios de rehabilitación con acceso equitativo a la población que se encuentra en desventaja física, económica y geográficamente remota, e incluye la evaluación, el monitoreo, la prevención, la intervención, la supervisión, la educación, la consulta y el asesoramiento de profesionales 10.

Las aplicaciones móviles de apoyo a la salud son herramientas necesarias en el día a día; la fisioterapia requiere un mayor énfasis en la utilización de contenidos digitales para el apoyo en la rehabilitación de la población 11-13. Esta alternativa no pretende sustituir a los profesionales, sino complementar y apoyar de manera más eficiente la atención sanitaria y facilitar el seguimiento a distancia 14.

El uso de aplicaciones requiere la participación activa del usuario, el compromiso en el buen uso y la corresponsabilidad sobre su condición de salud. Estas características hacen referencia al concepto de autogestión 7,12,15, el cual promueve la creatividad y la innovación en las estrategias de intervención y constituye una estrategia de apoyo al tratamiento convencional que disminuye los costos de los desplazamientos 16. Para la sociedad, la implementación de las TIC en la rehabilitación constituye una alternativa para influir en el comportamiento de las personas y promover el autocuidado. Además, se puede adaptar a las expectativas de los usuarios y permite equidad en el acceso 8,17,18.

En este sentido, se diseñó un protocolo de rehabilitación para el paciente amputado de miembro inferior en fase preprotésica y protésica, que sirvió como insumo para la creación de un software de fácil acceso mediante tecnologías móviles, que incluye animaciones, audioguías y texto con lenguaje sencillo.

El protocolo de intervención se enmarca en el modelo de atención integral y realiza acciones efectivas que impactan positivamente la salud y la calidad de vida del paciente amputado 9,19,20. El protocolo de intervención fisioterapéutica se diseñó de acuerdo con las necesidades del usuario y la evidencia científica disponible e incluyó el entrenamiento de cualidades físicas como la flexibilidad, la fuerza y el equilibrio, así como la reeducación de la sensibilidad, la propiocepción y el uso correcto del vendaje que ayuda a dar forma al muñón, condición básica para la protetización 21.

## METODOLOGÍA

La presente investigación tiene un enfoque metodológico cuantitativo y descriptivo, con el objetivo de diseñar y validar protocolos de rehabilitación fisioterapéutica basados en cinesiterapia activa para personas amputadas de miembro inferior en fase preprotésica y protésica.

En la etapa inicial para el diseño del protocolo se hizo la búsqueda en la literatura y posteriormente la validación del contenido por expertos en el área. La información que se analizó se recolectó de bases de datos como Google académico, PEDro, Pubmed, Scielo, BMC, Wolter Kluwer y Science Direct, además de artículos de revistas de salud editadas por Elsevier, Springer y SAGE Journal.

La estrategia de búsqueda incluyó palabras clave como amputación, amputación traumática, fisioterapia, terapia por ejercicio, medicina física y rehabilitación, rehabilitación, miembros artificiales, propiocepción, sensación, fuerza muscular, telerrehabilitación, adulto, anciano y actividades de la vida diaria, que se encuentran en los descriptores de ciencias de la salud DeSC y MeSH. De igual forma, se hizo la consulta en Google Scholar para la revisión de la normatividad (Ley 1751 de 2015, Política de Atención Integral en Salud (PAIS), Modelo de Acción Integral Territorial (MAITE), Ruta Integral de Atención en Salud y de Rehabilitación Funcional para Víctimas de MAP/MUSE (etapa 4), Fisioterapia Digital: Lineamientos y Prospectiva 11, Resolución 2654 de 2019).

Con base en la revisión bibliográfica se diseñó el protocolo de rehabilitación para personas con amputación de miembro inferior en fase preprotésica y protésica. El protocolo de rehabilitación es de tipo progresivo e incluye las cualidades físicas básicas del movimiento. Cada sesión tiene una duración de una hora, con una frecuencia de tres sesiones por semana, durante 10 semanas. Además, cuenta con una fase de sensibilización y cuidados del muñón, calentamiento, estiramiento, fase activa y enfriamiento.

Se hizo una convocatoria por escrito a trece expertos en el área de rehabilitación. Adjunt0 a la carta se envió la declaración de confidencialidad que soportó la participación voluntaria y la completa reserva de los documentos del estudio. Los expertos que aceptaron participar recibieron un resumen del proyecto, que incluyó el objetivo y la metodología de la investigación, y por medio de un formato construido por los investigadores se evaluó el contenido de los protocolos de rehabilitación definidos en la fase inicial del proyecto, en términos de adecuación, pertinencia y relevancia. Una vez surtida esta etapa se convocó a un consenso para definir los protocolos que se usaron en el software.

El grupo de expertos estuvo conformado por fisioterapeutas afiliados a la Asociación Colombiana de Fisioterapia, con experiencia en el área de conocimiento mínimo de cinco años, especialización en ortopedia y traumatología, así como con una experiencia superior a cinco años en la rehabilitación del paciente amputado, además de fisioterapeutas con especialización en rehabilitación cardiopulmonar y en seguridad y salud en el trabajo.

El instrumento de índice de validez de contenido global constó de 13 ítems, cuatro de ellos para adecuación, que hacían referencia a instrucciones fáciles de comprender, imágenes claras, número de series y repeticiones de acuerdo con las características de la población y la complejidad de los ejercicios. Para la pertinencia, los cuatro ítems evaluaban el plan de entrenamiento, la estructura del protocolo, los ejercicios de acuerdo con los niveles de amputación seleccionados y la progresión de los ejercicios y los cinco ítems de relevancia estaban relacionados con la percepción acerca del protocolo y las fases de calentamiento, estiramiento, fase activa y enfriamiento.

Para la validez de contenido se utilizó una escala tipo Likert con puntuación de 1 a 5, en la cual 1 se refiere a la más baja calificación y 5 a la más alta. Estos puntajes luego fueron traducidos a tres categorías, donde 1 y 2 se convirtieron en "no importante", 3 y 4 en "útil, no esencial", y 5 en "esencial", para el cálculo del índice de validez de contenido. Se estimó el coeficiente Kappa de Fleiss para cada criterio, donde para la interpretación se tuvo en cuenta la escala establecida por Landis 22, la cual expresa cualitativamente la fuerza de concordancia entre los evaluadores.

El protocolo definitivo se dispuso en un software para teléfonos móviles llamado Apptivate, diseñado mediante un trabajo interprofesional entre ingeniería de sistemas, encargados del diseño y la programación del software de la aplicación, diseño gráfico con la creación del contenido multimedia y fisioterapia con la construcción de los protocolos de rehabilitación. En el software se encuentra disponible la información del número de repeticiones y de series que se deben realizar, asimismo, cuenta con animaciones, texto y audio descriptivo de la ejecución de cada ejercicio como apoyo a personas con limitaciones auditivas y visuales.

## RESULTADOS

**Figura 1:**
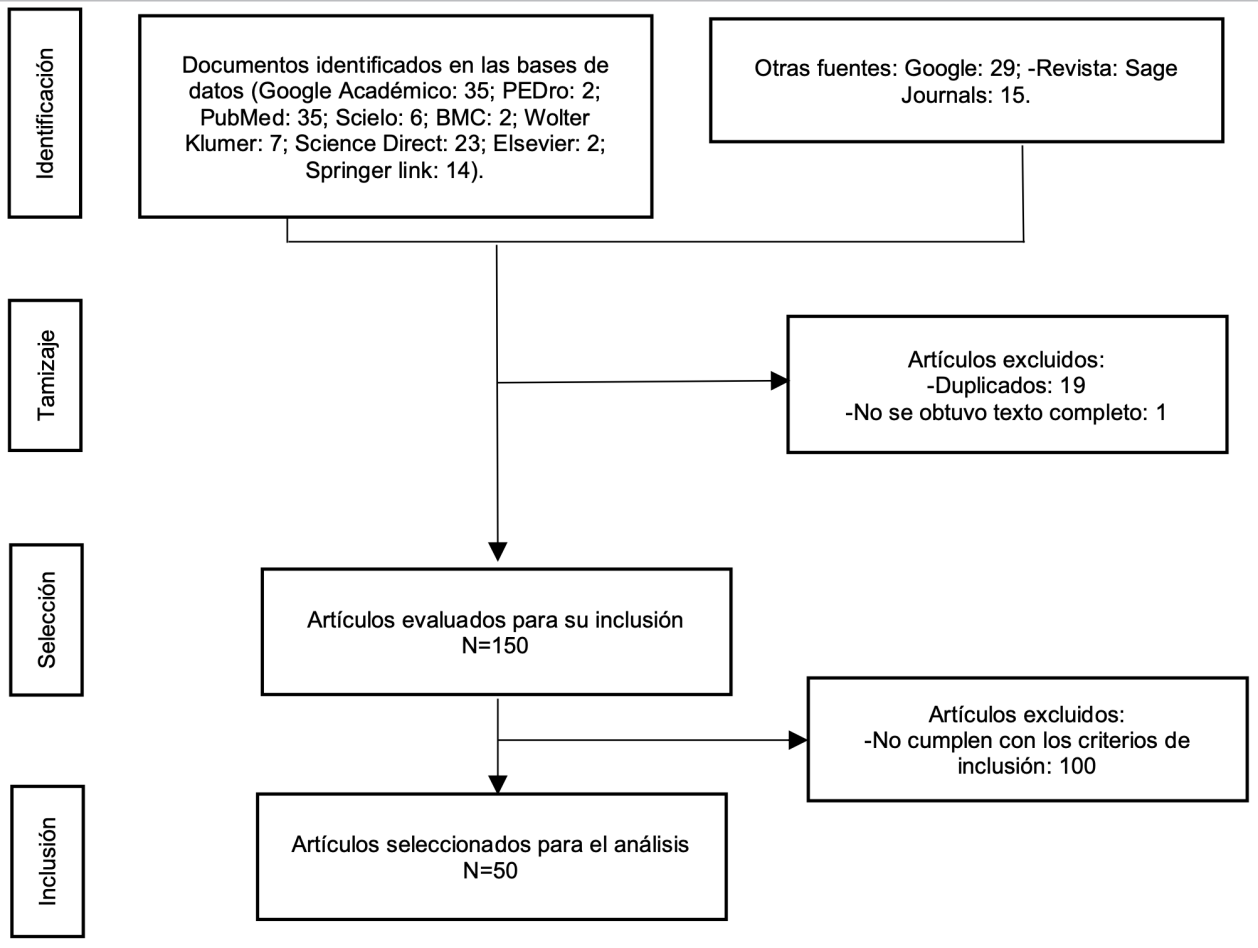
Diagrama de flujo prisma revisión de documento

En este estudio se revisaron 170 artículos y se seleccionaron 50 como objeto de investigación, que cumplieron con criterios de inclusión tales como investigaciones realizadas entre los años 2006 y 2020 publicadas en revistas y bases de datos indexadas, con rigor científico, carácter lógico, claridad y precisión en el contenido; investigaciones con población amputada de miembro inferior unilateral a nivel transfemoral, transtibial o desarticulación de rodilla relacionadas con la intervención fisioterapéutica en las fases preprotésica y protésica (Figura 1).

A nivel terapéutico, uno de los principales hallazgos fue que la técnica de facilitación neuromuscular propioceptiva tiene mejor resultado sobre las habilidades locomotoras y la fuerza de los músculos de la rodilla que el entrenamiento tradicional. En cuanto a los principales hallazgos en la revisión documental para la fase protésica, se destacó que la intervención interprofesional en el proceso de rehabilitación es fundamental para el mejoramiento de la calidad de vida del paciente y que la implementación de un programa de rehabilitación de ejercicios físicos mejora considerablemente el equilibrio y la marcha en los pacientes, siendo más efectivo que el tratamiento convencional.

**Figura 2:**
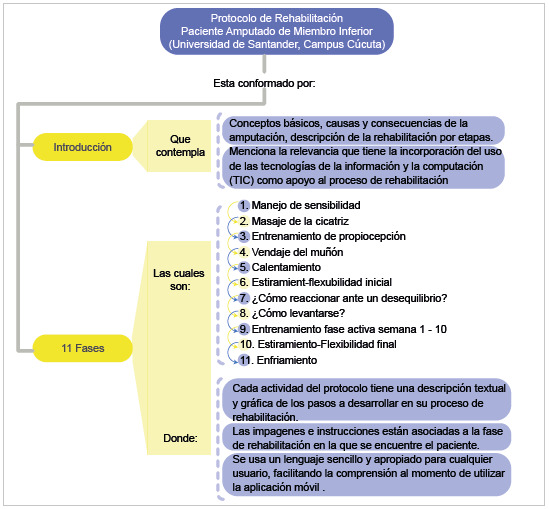
Protocolo de rehabilitación para las personas con amputación de miembro inferior

La construcción del protocolo de rehabilitación (Figura 2) se basó en la revisión documental y en los lineamientos de la OMS, así como en el aporte de expertos. El protocolo elaborado cuenta con una parte introductoria en la cual se aclaran algunos conceptos básicos, causas y consecuencias de la amputación y la descripción de la rehabilitación por etapas. El objetivo de la fase preprotésica es optimizar el miembro residual mediante la potenciación de las cualidades físicas.

Por otro lado, el objetivo de la fase protésica es lograr la independencia funcional por medio de cinesiterapia activa, entrenamiento del equilibrio estático y dinámico, transferencias de cargas y entrenamiento de la marcha en diferentes terrenos.

Se evidenció la importancia de incorporar el uso de las TIC como apoyo al proceso de rehabilitación, puesto que se logró una actitud más activa por parte del usuario, lo que implica la autogestión en el proceso de rehabilitación y refuerza la responsabilidad sobre su salud.

La validación de los protocolos de rehabilitación fue llevada a cabo por expertos en el área. Se utilizó el coeficiente Kappa de Fleiss para cada criterio, que expresa cualitativamente la fuerza de concordancia entre los evaluadores.

El cálculo de la validez de contenido *(Content Validity Ratio-cvR)* para cada ítem es:




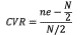




Donde: ne = el número de jueces que tienen acuerdo en la categoría "Escencial" y N = el número total de jueces.

La expresión anterior se plantea con la finalidad de interpretar los resultados como una correlación, por tomar valores de -1 a +1. De esta manera, CVR es negativa si el acuerdo ocurre en menos de la mitad de los evaluadores; es nula si se tiene exactamente la mitad de acuerdos en los expertos; y es positiva si hay más de la mitad de acuerdos 23.

A partir de la CVR calculada para todos los ítems, y aceptando aquellos que cuentan con valores superiores a los mínimos propuestos por Lawshe, se calcula la media de CVR, con lo que se obtiene el índice de validez de contenido de toda la prueba *(Content Validity Index-*CVI), y que se debe interpretar como la concordancia entre la capacidad solicitada en un dominio específico y el desempeño solicitado en la prueba que trata de medir dicho dominio 23. La expresión para el CVI es:




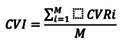




Donde: CVRi = la razón de validez de contenido de los ítems aceptables de acuerdo con el criterio de Lawshe, y M = el total de ítems aceptables de la prueba.

En la Tabla 1 se presentan los resultados para cada uno de los criterios evaluados, según el coeficiente Kappa de Fleiss con su respectivo intervalo de confianza.

**Tabla 1:** Coeficiente Kappa de Fleiss por cada criterio evaluado

Criterio	Coeficiente Kappa de Fleiss	IC 95%		Valor p	Concordancia
		Límite inferior	Límite superior		
Adecuación	0,420	0,320	0,520	0,000	Moderada
Pertinencia	0,429	0,324	0,534	0,000	Moderada
Relevancia	0,559	0,466	0,652	0,000	Moderada

Con base en la escala de medición ordinal, se encontró una fuerza de concordancia moderada para cada criterio, siendo el criterio de "relevancia" el más elevado (0,559) según el grado de acuerdo entre los profesionales de fisioterapia. Todos los coeficientes estimados fueron estadísticamente significativos (p<0,05).

Con relación a los resultados obtenidos para la razón de validez de contenido de cada ítem, como se presenta en la tabla 2, así como el cálculo del índice de validez de contenido del protocolo, se evidenciaron dos ítems con valoración máxima de validez y cinco ítems con valoración aceptable (CVR >0,60).

**Tabla 2:** Índice de validez de contenido global

Criterio	Ítem	Categorías			CVR
		No importante	Util, no esencial	Esencial	
Adecuación	1.- Las instrucciones que contiene el protocolo son fáciles de comprender		3	10	0,538
	2.- Las imágenes que acompañan las indicaciones son claras		2	11	0,692
	3.- El número de series y repeticiones está acorde a las características de la población que participa en el estudio		9	4	-0,385
	4.- La complejidad de los ejercicios se va incrementando de acuerdo con la etapa del protocolo (inicial, intermedia, final)		13		-1,000
Pertinencia	1. - El plan de entrenamiento tiene en cuenta las recomendaciones que se encuentran en la evidencia disponible		12	1	-0,846
	2. - La forma en la que se estructura el protocolo favorece el cumplimiento de los objetivos establecidos		4	9	0,385
	3. - El protocolo es específico a los niveles de amputación para los que fue diseñado		2	11	0,692
	4. - Los ejercicios del protocolo son progresivos, se evidencia un incremento en el nivel de complejidad		1	12	0,846
Relevancia	1.- Considera que la aplicación del protocolo mejora la condición física del paciente (sensibilidad, manejo de la cicatriz, propiocepción, aprender a caer y levantarse, fuerza, equilibrio, marcha)		1	12	0,846
	2.- Considera que es importante la fase de calentamiento para lograr una preparación del organismo antes de iniciar la fase de entrenamiento			13	1,000
	3.- Considera que es importante la fase de estiramiento para elongar las estructuras implicadas y adyacentes con el propósito de evitar lesiones		11	2	-0,692
	4.- Considera que es importante la fase de enfriamiento para normalizar funciones orgánicas y el equilibrio homeostático general después de realizar la fase entrenamiento			13	1,000
	5.- Considera que la aplicación del protocolo mejora la funcionalidad del paciente		1	12	0,846
	Sumatoria (todos los ítems)				3,923
	Índice de validez de contenido (todos los ítems)				0,302
	Sumatoria (ítems aceptables)				5,923
	Índice de validez de contenido (ítems aceptables)				0,846

De acuerdo con los resultados obtenidos, se evidenció un índice de validez de contenido de 0,846, lo cual indica que el protocolo tiene propiedades aceptables para ser utilizado como herramienta de intervención del paciente amputado de miembro inferior.

## DISCUSIÓN

En el año 2016, Whitehead 9 evaluó la efectividad del uso de aplicaciones móviles en el manejo de diferentes condiciones de salud, haciendo énfasis en que son una estrategia innovadora que permite mayor acceso a los servicios de salud y oportunidad en la atención; además, fortalece, la autogestión del usuario, lo que implica un mejor resultado de las intervenciones.

Entretanto, Villarreal 24 realizó un estudio cuyo objetivo fue integrar tecnologías móviles para mejorar la cobertura y la asistencia a los servicios de salud con el propósito de reducir costos y tiempo sin reemplazar las funciones del fisioterapeuta. En la medida en la que se inicia tempranamente la intervención se previenen complicaciones, lo que permite reducir costos en la atención. El uso de la telerrehabilitación disminuye la brecha de la falta de cobertura de los servicios de salud en regiones apartadas y además es una estrategia de apoyo al tratamiento convencional.

Por su parte, Carvalho 25 diseñó una aplicación móvil con la metodología de diseño centrado en el usuario (DCU), que busca la integración entre usuarios y diseñadores en la fase inicial de concepción de la aplicación. El presente estudió incluyó profesionales en las áreas de fisioterapia, ingeniería de sistemas y diseño gráfico, quienes basaron el desarrollo de la aplicación en la metodología de diseño centrado en el usuario.

Webster 26, por su parte, publicó las guías de práctica clínica para la rehabilitación de amputados de miembros inferiores, haciendo énfasis en la educación del paciente y del cuidador con el fin de mejorar la calidad de la atención en la rehabilitación para personas amputadas. Los protocolos diseñados en esta investigación involucran la participación de un familiar que realice acompañamiento al paciente en aras de evitar la presentación de eventos adversos durante la ejecución del plan de intervención.

Greitemann 27 llevó a cabo un estudio cuyo objetivo fue establecer un protocolo de tratamiento para la rehabilitación de pacientes amputados de miembro inferior, para lo cual se basó en la fase preoperatoria, que incluye ejercicios de cinesiterapia, y la fase postoperatoria, que incluye ejercicios activos asistidos, libres y resistidos, determinantes en el fortalecimiento, el entrenamiento de la marcha y en mejorar la destreza en la ejecución de las actividades de la vida diaria. Este estudio es similar al protocolo propuesto en esta investigación que contiene ejercicios de cinesiterapia, entrenamiento de marcha, equilibrio, fuerza y flexibilidad, con el propósito de mejorar la funcionalidad en el paciente.

En el Hospital Militar Central de Bogotá, Colombia 7, en el año 2015 se diseñó una guía de manejo para la rehabilitación del paciente amputado de miembro inferior, documento en el que intervinieron diferentes especialidades como medicina física, ortopedia, terapia física, terapia ocupacional y psicología, tanto en el manejo preprotésico como protésico. Se encontró similitud con este estudio ya que los dos protocolos involucran actividades que aumentan su nivel de complejidad de manera progresiva, permitiendo la adaptación del usuario, al tiempo que se evitan riesgos y se consigue la reincorporación de los participantes en los roles familiar, laboral y social.

En la guía de manejo titulada "Los amputados y su rehabilitación, un reto para el Estado mexicano" 4, del año 2016, se hace el énfasis en la carencia de instituciones gubernamentales encargadas de la rehabilitación de los pacientes amputados de miembro inferior, situación similar a la que se presenta en Colombia. El Gobierno Nacional de Colombia diseñó la ruta integral de atención en salud para la rehabilitación funcional de víctimas de minas antipersona y municiones sin explosionar, con la finalidad de dar respuesta a una necesidad sentida de la población. Esta ruta se basa en la normatividad vigente (Ley 100 de 1993), la cual establece que "el sistema general de seguridad social en salud brindará atención en salud integral a la población en sus fases de educación, información y fomento de la salud y la prevención, diagnóstico, tratamiento y rehabilitación, con oportunidad, calidad y eficiencia".

En otro estudio similar, realizado por Pierotti 28 en el año 2020, diferentes expertos validaron mediante un instrumento el protocolo de intervención. Los expertos que hicieron parte de este proceso tenían un tiempo de experiencia mayor a cinco años en el área y conformaban un equipo interprofesional que abordaba la condición del usuario desde un enfoque biopsicosocial. Estas características cualifican a los expertos como profesionales idóneos para llevar a cabo la validación, teniendo en cuenta los criterios de adecuación, pertinencia y relevancia. Lo anterior permitió comprobar que el protocolo propuesto tiene propiedades aceptables para ser utilizado como herramienta de intervención del paciente amputado de miembro inferior.

Entre las limitaciones evidenciadas en el proceso se encuentra la selección de los expertos en las diferentes áreas, la disponibilidad y la aceptación para participar en el estudio, además las diversas perspectivas e interpretaciones hacen subjetiva la validación del protocolo.

Se llevaron a cabo cinco encuentros entre investigadores y expertos, en los cuales se aclararon aspectos relacionados con la estructura del protocolo y el aumento en la complejidad de los ejercicios, de conformidad con la semana de intervención. Lo anterior permitió hacer ajustes en el diseño del protocolo para lograr una fácil interpretación por parte de los usuarios.

Los resultados obtenidos en la presente investigación son el insumo para la segunda fase del proyecto, en la cual se llevará a cabo la implementación del protocolo dispuesto en el software con el fin de determinar la efectividad de este tipo de intervenciones, además de identificar aspectos susceptibles de modificación para tener una versión más intuitiva de la aplicación.

Se evidenció, por medio de la validación por expertos, que el protocolo de rehabilitación para la fase preprotésica y protésica tiene propiedades aceptables para ser utilizado como herramienta de intervención del paciente amputado de miembro inferior, ya que representa un manejo integral del usuario y hace énfasis en la funcionalidad para lograr la reincorporación a los diferentes roles.

La telerrehabilitación se convierte en una estrategia de intervención novedosa en el campo de la fisioterapia que utiliza las tecnologías de la información y la comunicación para llegar a la población que tiene dificultades para acceder a los servicios de salud, y a su vez representa una alternativa complementaria al proceso de rehabilitación convencional.

Se logró evidenciar que la implementación de las TIC en la rehabilitación incentiva el proceso de autogestión sobre la condición de salud por parte del usuario, siendo este un aspecto diferenciador con el que se logran mejores resultados que en el tratamiento convencional, en el cual el fisioterapeuta asume toda la responsabilidad.

Para las instituciones de educación superior, es importante incluir en el currículo el uso de las TIC en salud para formar un talento humano competente para dar respuesta a las necesidades identificadas en la población.

Para el gobierno, es necesario ampliar la infraestructura tecnológica que permita el acceso a los servicios de telerrehabilitación en la población que tiene dificultades para acceder a los servicios de salud.

Es necesario educar al paciente acerca de la importancia de la autogestión en su condición de salud, con el fin de obtener un mejor resultado en el proceso de rehabilitación.

Es importante incentivar que los fisioterapeutas incluyan el uso de las TIC como una estrategia complementaria a la rehabilitación convencional, lo cual permitirá evidenciar su compromiso ético y de responsabilidad social.

Este estudio se desarrolló como parte del proyecto marco "Modelo de apoyo a la rehabilitación del paciente amputado de miembro inferior basado en concepto de autogestión del usuario utilizando tecnologías móviles en instituciones de salud de la ciudad de Cúcuta".

La fuente de financiación de este estudio fue en especie y correspondió a profesionales de fisioterapia, ingeniería de sistemas y diseño gráfico de la Universidad de Santander campus Cúcuta y un ingeniero mecatrónico del Servicio Nacional de Aprendizaje (SENA) ♠
